# Effect of smoking and soft tissue release on risk of revision after total knee arthroplasty: a case- control study

**DOI:** 10.1186/s12891-015-0694-z

**Published:** 2015-09-09

**Authors:** Benedict U. Nwachukwu, Ellen B. Gurary, Vladislav Lerner, Jamie E. Collins, Thomas S. Thornhill, Elena Losina, Jeffrey N. Katz

**Affiliations:** Department of Orthopedic Surgery, Boston, USA; Division of Rheumatology, Immunology and Allergy, Boston, USA; The Department of Orthopedic Surgery, Hospital for Special Surgery, New York, USA; Department of Orthopedic Surgery, Massachusetts General Hospital, Boston, USA; Department of Biostatistics, Boston University School of Public Health, Boston, USA; Orthopedic and Arthritis Center for Outcomes Research, Brigham and Women’s Hospital, 75 Francis Street, BC-4016, Boston, MA 02115 USA

## Abstract

**Background:**

Increasing utilization of primary total knee arthroplasty (TKA) is projected to expand demand for revision TKA. Revision TKAs are procedurally complex and incur high costs on our financially constrained healthcare system. The purpose of this study was to use a case-control design to identify factors predisposing to revision TKA, particularly demographic, clinical and perioperative technical factors.

**Methods:**

We conducted a case control study to investigate patient, surgical and perioperative factors associated with greater risk of revision TKA. We included patients who received TKA at a tertiary center between 1996 and 2009. Cases (patients that had primary and revision TKA) were matched to controls (patients with primary TKA that was not revised) in a 1:2 ratio and risk of revision examined using conditional logistic regression.

**Results:**

We identified 146 cases and 290 controls. Patient factors independently associated with revision included male sex (OR 1.73; 95 % CI 1.06-2.81) and smoking (OR 2.87; 1.33-6.19). Older age was associated with decreased risk (OR 0.83 per 5-year increment; 95 % CI 0.75-0.92). Lateral release was the only technical factor associated with revision (OR 1.92; 1.07-3.43).

**Conclusions:**

In this case control study younger patient age, male gender, soft tissue release and active smoking status were associated with increased revision risk. Although we do not know whether the risk of smoking arises from short- or long-term exposure, smoking cessation prior to TKA should be considered as an intervention for decreasing revision risk.

## Background

Total Knee Arthroplasty (TKA) is an effective surgical intervention for the relief of the symptoms and functional disability associated with advanced knee arthritis [1]. More than 610,000 primary total knee replacements were performed in 2012 incurring aggregate costs exceeding $9.8 billion [2]. The increasing utilization of primary TKA is projected to result in substantial increases in demand for revision TKA, which was performed on over 54,000 persons in the US in 2012 [2]. Recent evidence suggests that the utilization of TKA is growing fastest in younger patients [3], a population at higher risk for revision procedures [4]. Given the procedural complexity of revision procedures, growth in utilization of revision TKAs will further strain healthcare resources [5].

The growing use of primary TKA and attendant burden of revision TKA have heightened interest in identifying risk factors for revision. Several factors have been reported to be associated with an elevated risk for revision TKA including male sex [6–8], younger age [6, 8], medical comorbidity [8], socioeconomic status [8], surgical technique [9, 10], body mass index (BMI) [11], post-operative knee alignment [11] and prior knee surgery [12]. The literature is limited, however, because many papers rely on administrative/registry databases [13, 14], which often do not have rich clinical detail to analyze for patient and technical factors associated with revision.

Given the limitations in the current evidence base, the purpose of this study was to use a case-control design to identify factors predisposing to revision TKA, particularly demographic, clinical and perioperative technical factors.

## Methods

### Study design

We conducted a case–control study of risk factors for revision TKA after primary TKA.

### Selection of patients

#### Patient population

We identified subjects using the Partners Research Patient Data Registry (RPDR), a data repository that permits identification of all procedures performed at Brigham and Women’s Hospital (BWH), Boston, MA USA using *International Classification of Diseases Ninth Edition* (ICD-9) and Current Procedural Terminology (CPT) codes. Patients were eligible for inclusion if they received a TKA between January 1996 and January 2009 at the BWH. The Institutional Review Board (IRB) at the BWH approved this study and the study design was exempt from the requirement for informed consent.

#### Identification of cases and controls

Patients were eligible for inclusion as cases if they underwent both primary TKA *and* subsequent revision TKA at BWH. We attempted to match each case with a control patient who received primary TKA at BWH and did not undergo revision TKA during the study period. We matched cases to controls in a 1:2 ratio based on primary surgeon and year of surgery. Matching by surgeon was done to eliminate the risk of revision attributable to surgeon factors and matching by year was done to minimize the impact of changing surgical techniques over the time period of the study.

#### Chart review

Following the identification of cases and controls we conducted a medical record review. We obtained medical record information from the RPDR, paper medical record or BWH electronic Longitudinal Medical Record (LMR). Specific data elements that we abstracted included: primary underlying joint disease, medical comorbidities (past medical history, medications, body mass index), prior orthopaedic and non-orthopaedic surgeries, socio-demographics (employment status, living conditions, marital status, ambulatory status and smoking status), perioperative factors (hospital length of stay, discharge destination, antibiotic treatment), surgical factors (anesthesia type, primary surgeon, surgical and tourniquet time, surgical approach, fixation type, ligament/soft tissue releases), implant type and design, pre and post-operative radiographic alignment and immediate post-operative complications. We abstracted data on patient and technical factors for inclusion in analysis.

For subjects undergoing revision TKA the medical record review also included indication for revision, revision procedure performed, operative findings and primary surgeon.

### Statistical analysis

We used conditional logistic regression for matched data to calculate univariate odds ratios characterizing the association between covariates and case/control status, and 95 % confidence intervals (CI). We advanced factors associated with revision with *p*-value less than 0.1 or odds ratio greater than 1.5 or less than 0.75 from univariate analysis to multivariable conditional logistic regression analysis. Univariate associations are presented as crude odds ratios and multivariate associations as adjusted odds ratios based on the final model. Because there was some missing data on whether a lateral release was performed, we introduced an indicator variable “lateral release missing” to examine revision risk in subjects with missing data on lateral release and to avoid dropping these patients from the model. The sample size of 146 cases generally supports a multivariate analysis of ~ 14 risk factors [15, 16]. We performed a pair of sensitivity multivariate analyses in which we first restricted the sample to cases due to infection (and their controls) and then to cases due to aseptic revision (and their controls).

## Results

### Sample

Our study includes a total of 147 consecutive revision TKAs (cases). One case could not be matched with a control TKA and was excluded, leaving 146 cases in the sample. Of these cases, 144 had two controls and 2 could only be matched to one control yielding a total of 290 controls. Thus the final sample included 146 cases and 290 controls.

The mean age of the cases was 57.8 (sd.13.2) years and 65.4 (sd.12.6) years for the controls. The majority of cases (*N* = 91; 62 %) and controls (*N* = 216; 74 %) were female. Osteoarthritis (OA) was the predominant indication for primary TKA in cases (*N* = 118; 84 %) and controls (*N* = 266; 92 %) (Table 1).

**Table 1:** Case control comparison for risk of revision based on demographic and technical factors

Variables	Cases (*N* = 146)	Controls (*N* = 290)	Crude odds ratios	Adjusted odds ratios- final model^a^
Continuous	Mean (SD)	Mean (SD)	OR (95 % CI)	OR (95 % CI)
Age at Surgery ^b^	57.8 ± 13.2	65.4 ± 12.6	0.79 (0.72, 0.86)	0.83 (0.75, 0.92)
Cardiac Risk (0,1,2+)^c^	1.6 ± 0.7	1.7 ± 0.7	0.91 (0.67, 1.22)	
Categorical	N (%)	N (%)	OR (95 % CI)	OR (95 % CI)
Gender				
Male	55 (38 %)	74 (26 %)	1.77 (1.15,2.72)	1.73 (1.06, 2.81)
Female	91 (62 %)	216 (74 %)	reference	reference
Smoking				
Yes	29 (22 %)	18 (6 %)	4.46 (2.21, 9.03)	2.87 ( 1.33, 6.19)
No	104 (78 %)	263 (94 %)	reference	reference
OA Diagnosis at Primary				
No	22 (16 %)	24 (8 %)	2.03 (1.09, 3.79)	1.55 (0.75, 3.22)
Yes	118 (84 %)	266 (92 %)	reference	Reference
BMI Category				
<30	75 (51 %)	153 (53 %)	reference	
30–35	31 (21 %)	70 (24 %)	0.84 (0.52,1.35)	
35+	40 (27 %)	67 (23 %)	0.77 (0.44, 1.34)	
Diabetes				
Yes	14 (10 %)	43 (15 %)	0.62 (0.33, 1.17)	
No	132 (90 %)	247 (85 %)	reference	
PCL Recession				
Yes	19 (15 %)	51 (19 %)	0.67 (0.35, 1.26)	
No	111 (85 %)	212 (81 %)	reference	
Lateral Release				
Yes	42 (29 %)	59 (20 %)	1.85 (1.13, 3.01)	1.92 (1.07, 3.43)
No	88 (60 %)	216 (75 %)	Reference	Reference
Missing	16 (11 %)	15 (5 %)	2.61 (1.24, 5.47)	1.68 (0.68, 4.13)
Post-Op Return to OR				
Yes	22 (15 %)	10 (3 %)	4.65 (2.13, 10.13)	
No	124 (85 %)	280 (97 %)	reference	

^a^Final model only includes final variables meeting the p ≤ .1 criterion

^b^Odds Ratios in units of 5 years

^c^The following were defined as cardiac risk factors: Coronary Artery (CAD); Coronary Artery Bypass Graft (CABG); Atherosclerotic Cardiovascular (ASCVD); Ischemic Heart Disease. Patients were assigned a score (0,1,2+) based on a sum for each individual disease (0 if no disease and 1 if disease was present)

Infection was the most common indication for revision (*N* = 44, 30 %), followed by aseptic loosening (*n* = 26, 18 %) and stiffness (*n* = 26, 18 %) and then instability (*n* = 19, 13 %) (Table 2).

**Table 2:** Indications for revision^a^

Causes of Failure	Total
	*N* = 146
Aseptic Loosening	26 (18 %)
Extensor Mechanism Failure	2 (1 %)
Infection	44 (30 %)
Instability	19 (13 %)
Periprosthetic Fracture	2 (1 %)
Polyethylene Liner Wear	1 (1 %)
Repeated Dislocation	3 (2 %)
Stiffness	26 (18 %)
Subsidence	2 (1 %)
Swelling	10 (7 %)
Other	11 (8 %)

^a^Indications listed hierarchically. If subject had more than one indication, he or she was assigned the one highest on this list

### Univariate analysis of factors associated with revision

In univariate analysis cases were more likely to be younger than controls (OR 0.79 per 5 years; 95 % CI 0.72-0.86). Males had a statistically significant increased risk for revision (OR 1.77; 95 % CI 1.15-2.72). Receiving TKA for a non-OA diagnosis increased the risk of revision (OR 2.03; 95 % CI 1.09-3.79). Twenty-nine cases (22 %) and 18 controls (6 %) were current smokers. Active smoking status was significantly associated with revision (OR 4.46; 95 % CI 2.21-9.03) (Table 1). We did not observe statistically significant associations between revision and cardiovascular comorbidity, diabetes, pre-operative varus/valgus malalignment and pre-operative ambulatory status.

With regard to technical factors, intra-operatively, 29 % of cases received a lateral release at the time of primary TKA compared to 20 % of controls. The use of a lateral release significantly increased the risk of revision (OR 1.85; 1.13-3.01). Cases were also more likely to have missing data on lateral release than controls (Table 1). Further analysis showed that of the 42 cases that had lateral release, 18 (46 %) were revised for infection. PCL technique (sparing/sacrifice) did not have a statistically significant association with revision risk.

Factors that were not associated with revision risk (at *p* < 0.05 or 0.75 < OR < 1.5) are not shown on Table 2.

### Multivariate analysis of factors associated with revision

We performed multivariate, conditional logistic regressions analyses, advancing all variables with univariate *p*-values < 0.1 or odds ratios > 1.5 or < 0.75. The final model is shown in Table 1. We did not advance return to OR to the final model because information on return to OR is not known preoperatively and therefore cannot be incorporated into preoperative planning. In our final model increased age was associated with decreased revision risk (OR 0.83 per 5 years; 95 % CI 0.75-0.92); male sex (OR 1.73; 1.06-2.81) and current smoking status (OR 2.87; 1.33-6.19) were associated with increased revision risk. Lateral release (OR 1.92; 1.07-3.43) was associated with an increased risk of revision and having missing data on lateral release was also associated with an increased risk of revision, although this association did not reach statistical significance (OR 1.68, 0.68, 4.13).

We performed exploratory analyses in which we examined risk factors for revision due to infection and risk factors for aseptic revision in separate models. In these models, lateral release was associated strongly with risk of revision for infection (OR 5.28, 95 % CI 1.69, 16.47) but the data did not provide evidence for a clinically important relationship of lateral release with aseptic revision (OR 1.28, 95 % CI 0.60, 2.74). In contrast, smoking was associated strongly with risk of aseptic revision (OR 4.41, 95 % CI 1.67, 11.62) but the data did not support a clinically important relationship of smoking with risk for infectious revision (OR 1.22 95 % CI 0.23, 6.64). As in the models of the entire cohort, the adjusted ORs for the group of subjects missing data on lateral release were similar to those for lateral release.

## Discussion

We performed a case control study to investigate factors associated with revision TKA at a tertiary care center. Patient factors increasing the risk for revision included younger age, male gender and smoking status. Technical factors associated with increased revision risk included receiving a lateral release.

Our finding that males and younger patients have an increased risk for revision is a consistent finding in the literature and has been reproduced in large database studies [13, 8]. Curtin et al. [8] found, based on analysis of 61,767 TKAs, that younger male patients were more likely to require a revision procedure after primary TKA. This phenomenon is likely explained by greater stresses placed on the implant by young active male patients and potentially greater reluctance among clinicians to revise a TKA in older patients.

We also found that smoking status has a strong association with revision risk for TKA. While smoking has been associated with an increased risk of general post-operative complication after TKA [17–19], only one previous study has specifically examined the association between revision TKA and smoking status [20]. Kapadia et al. performed a survivorship analysis of TKAs performed on 531 patients at a mean follow-up of 47 months and found that any history of smoking (current or former) was associated with a higher revision rate (10 % in smokers vs 1 % in non-smokers). Our finding in a study with longer follow up and controlling for potential confounders confirms this association. Although the pathophysiology underlying the increased risk for revision TKA in smokers has not been clearly elucidated, cigarette smoke has been shown to disrupt osteointegration, a fundamental process in the longevity of implanted materials [21]. In addition, nicotine has been found to inhibit secretion of tumor necrosis factor (TNF) alpha—a critical cytokine for bone remodeling—through activation of the cholingergic anti-inflammatory pathway [22]. Previous evidence has shown that smoking cessation programs can attenuate the general surgical risk associated with active smoking status [23]. More attention should be paid to understanding the impact of smoking cessation on revision risk after TKA.

There is increasing interest in understanding the technical factors associated with revision TKA. In this study we found that performing a lateral release increased the risk for a revision TKA. Lateral release during TKA is a surgical technique whereby tight lateral soft tissue structures of the knee are released. Such releases are most often employed in valgus knees in order to balance the knee and restore joint kinematics. The impact of lateral release during TKA has been previously studied. Patellofemoral maltracking and patella osteonecrosis/fracture are potential biomechanical complications associated with lateral release. However there has been evidence to suggest that with appropriate surgical technique these complications are avoidable and do not increase revision risk [24, 25]. Similar to these prior studies our subgroup analysis did not find an increased incidence of instability or loosening in patients requiring a lateral release. We did find however that lateral release was associated with increased risk for infection related revision. A few prior studies have suggested that there is potentially an association between soft tissue releases and increased infection risk after TKA. Kumar and Dorr [26] hypothesized that in valgus knees requiring soft tissue release there is an increased risk for infection due to the creation of irregular tissue planes and dead space, which may serve as a nidus for infection. Johnson and Eastwood [27] similarly found that when the superolateral geniculate vessels are divided during lateral release there is an increased risk of wound infection and skin viability as assessed by skin oxygen tension. Lateral releases are an important part of knee balancing during TKA and should continue to be utilized when indicated however continued attention should be paid to understanding the risk factors for revision and the role of lateral releases. The risk for revision noted in the present study for lateral release is likely separate from that associated with having valgus alignment alone, since valgus alignment was not associated with revision risk in our analyses.

This study has several limitations. Our case control match was based on surgeon and year of primary procedure. Matching on surgeon precludes analysis of surgeon factors that may play a role in revision risk (e.g. arthroplasty fellowship training or surgeon volume). As part of this study we looked at the impact of pre-operative alignment however we did not look at the severity and degree of knee malalignment on revision risk; this may have modulated the impact of lateral release. Further as part of our dataset, operative information on lateral release was missing in a select number of our cases; this may have influenced our findings. Our assessment of smoking status in this study was also limited. We were unable to obtain information on pack-years and thus cannot comment on dose response or the effect of smoking cessation on revision risk. We also did not have data on alcohol or illicit drug use. Finally, previous studies have suggested that social and ethnic factors contribute to TKA outcome [28]. Due to inconsistent reporting of race and socioeconomic variables such as income or education within the database, and lack of information on payor status, we were not able to sufficiently track these variables to use in this study.

The major strength of this study is that we performed detailed medical record reviews. We used a detailed review of institutional medical records, with outpatient, in-patient and intra-operative data. This approach enabled us to identify factors such as smoking status and lateral release that generally cannot be determined from large administrative medical datasets but which appear to influence revision risk. More work needs to be done to understand the impact of lateral release on revision risk. Intra-operative lateral release may be indicative of other factors associated with increased likelihood for revision. For example, requiring a lateral release may be indicative of procedural complexity. Similarly, technical errors such as excessive femoro-tibial rotation may necessitate a lateral release, certain implants are associated with a higher incidence of lateral release and certain surgeons may perform these releases more commonly.

## Conclusion

In this case control study younger patient age, male gender, soft tissue release and active smoking status were associated with increased revision risk. Although we do not know whether the risk of smoking arises from short- or long-term exposure, smoking cessation prior to TKA should be considered as an intervention for decreasing revision risk. Lateral release is a technical factor associated with increased risk for revision after TKA.

## Abbreviations

BMI: Body mass index
OA: Osteoarthritis
TKA: Total knee arthroplasty
